# Supplementary material for: An ATP-gated molecular switch orchestrates human mRNA export
Source: Nature. 2025 Nov 6;649(8098):1042–50. doi: 10.1038/s41586-025-09832-z (PMC12823420; doi:10.1038/s41586-025-09832-z)
Supplement: Supplementary file 1 — Guide to Supplementary Tables 1–5 and Supplementary Fig. 1 (the uncropped blots). [file 41586_2025_9832_MOESM1_ESM.pdf]

nature portfolio

https://doi.org/10.1038/s41586-025-09832-z

# Supplementary information

# An ATP-gated molecular switch orchestrates human mRNA export

In the format provided by the
authors and unedited

# Supplementary Information

## An ATP-gated molecular switch orchestrates human messenger RNA export

Ulrich Hohmann[1,2,#,*], Max Graf[1,3], László Tirián[2], Belén Pacheco-Fiallos[1,3], Ulla Schellhaas[1,3], Laura Fin[1], Dominik Handler[2], Alex W. Philipps[1], Daria Riabov-Bassat[1], Rupert W. Faraway[1,4,5], Thomas Pühringer[1], Michael-Florian Szalay[1], Elisabeth Roitinger[1,2], Julius Brennecke[2,*], and Clemens Plaschka[1,*]

[1]*Research Institute of Molecular Pathology (IMP), Vienna BioCenter (VBC), 1030 Vienna, Austria.*

[2]*Institute of Molecular Biotechnology of the Austrian Academy of Sciences (IMBA), Vienna BioCenter (VBC), 1030 Vienna, Austria.*

[3]*Vienna BioCenter PhD Program, Doctoral School of the University of Vienna and Medical University of Vienna, 1030 Vienna, Austria*

[4]*Max Perutz Labs, Vienna BioCenter (VBC), Dr.-Bohr-Gasse 9, A-1030, Vienna, Austria*

[5]*University of Vienna, Max Perutz Labs, Department of Biochemistry and Cell Biology, Dr.-Bohr-Gasse 9, A-1030 Vienna, Austria*

[#]*Current address: Institute of Molecular Biology (IMB) gGmbH, 55128 Mainz, Germany*

**Correspondence to: u.hohmann@imb-mainz.de, julius.brennecke@imba.oeaw.ac.at, clemens.plaschka@imp.ac.at*

**Supplementary Data Tables, supplied as separate files:**

**Supplementary Data Table 1 | Proteomics data of native UAP56-GFP immunoprecipitation.**

**Supplementary Data Table 2 | Proteomics data of native TREX–mRNP disassembly assay.**

**Supplementary Data Table 3 | AlphaFold2 Multimer screen results for UAP56 and its putative interactors.**

**Supplementary Data Table 4 | Proteomics data of flag-UAP56 immunoprecipitations.**

**Supplementary Data Table 5 | Vectors and sequences.**

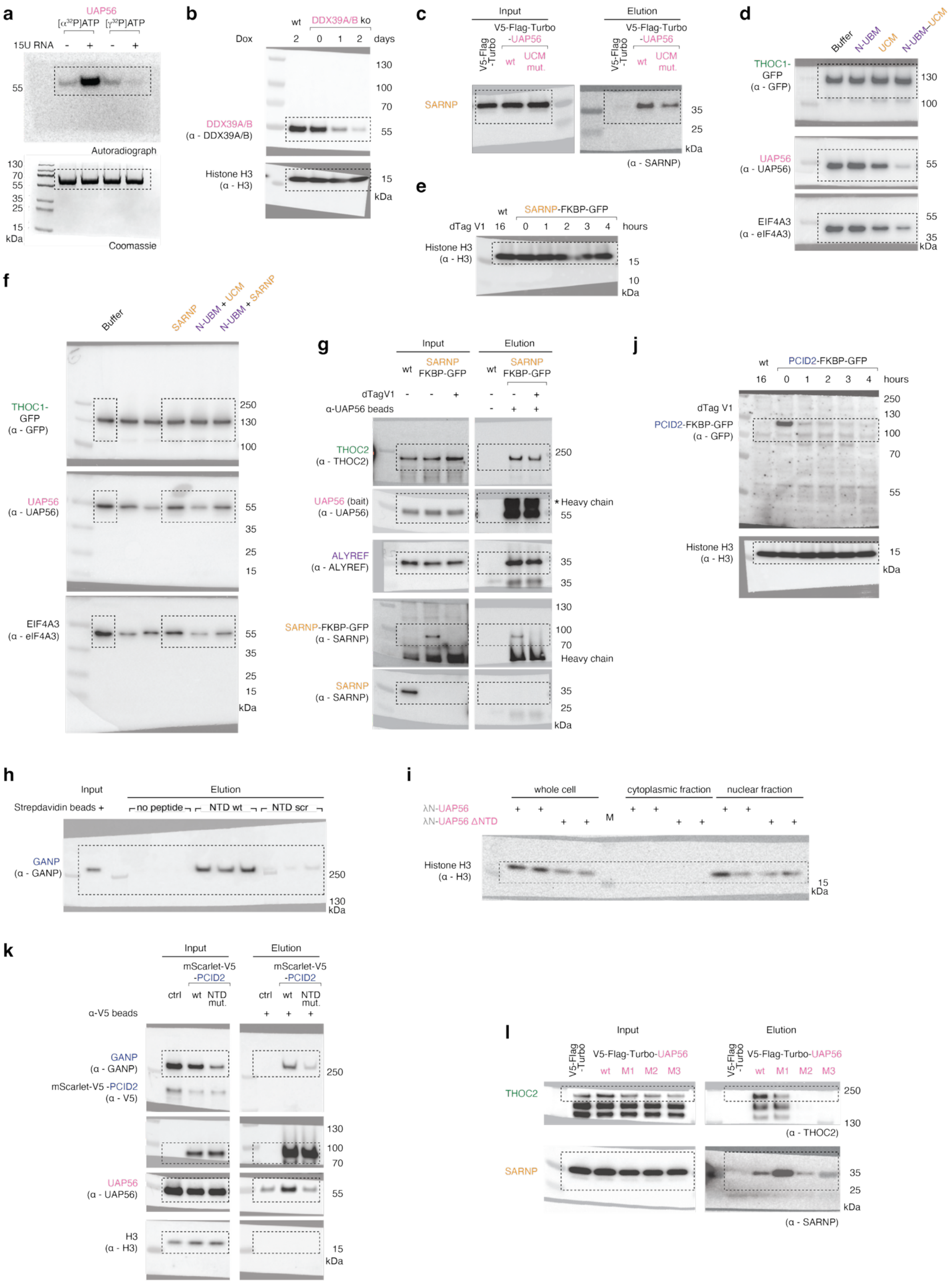


**Supplementary Figure 1 | Raw images for gels and western blots.**

Dashed lines indicate the regions shown in the respective Figure or Extended Data Figure.

**a,** Uncropped autoradiograph (top) and gel image (bottom) for Fig. 1h.

**b-l**, Uncropped western blots for Extended Data Figs 3b (**b**), 4d (**c**), 5e (**d**), 5g (**e**), 5f (**f**), 5h (**g**), 9g (**h**), 9j (**i**), 10a (**j**), 10e (**k**), 10l (**l**)
