# Supplementary material for: An ATP-gated molecular switch orchestrates human mRNA export
Source: Nature. 2025 Nov 6;649(8098):1042–50. doi: 10.1038/s41586-025-09832-z (PMC12823420; doi:10.1038/s41586-025-09832-z)
Supplement: Supplementary file 2 — Reporting Summary [file 41586_2025_9832_MOESM2_ESM.pdf]

nature research

Corresponding author(s): Plaschka

Last updated by author(s): Oct 23, 2025

Reporting Summary

Nature Research wishes to improve the reproducibility of the work that we publish. This form provides structure for consistency and transparency in reporting. For further information on Nature Research policies, see our Editorial Policies and the Editorial Policy Checklist.

Statistics

For all statistical analyses, confirm that the following items are present in the figure legend, table legend, main text, or Methods section.

n/a	Confirmed	
☐	☒	The exact sample size (*n*) for each experimental group/condition, given as a discrete number and unit of measurement
☒	☐	A statement on whether measurements were taken from distinct samples or whether the same sample was measured repeatedly
☐	☒	The statistical test(s) used AND whether they are one- or two-sided<br>*Only common tests should be described solely by name; describe more complex techniques in the Methods section.*
☒	☐	A description of all covariates tested
☒	☐	A description of any assumptions or corrections, such as tests of normality and adjustment for multiple comparisons
☐	☒	A full description of the statistical parameters including central tendency (e.g. means) or other basic estimates (e.g. regression coefficient) AND variation (e.g. standard deviation) or associated estimates of uncertainty (e.g. confidence intervals)
☐	☒	For null hypothesis testing, the test statistic (e.g. *F*, *t*, *r*) with confidence intervals, effect sizes, degrees of freedom and *P* value noted<br>*Give P values as exact values whenever suitable.*
☒	☐	For Bayesian analysis, information on the choice of priors and Markov chain Monte Carlo settings
☒	☐	For hierarchical and complex designs, identification of the appropriate level for tests and full reporting of outcomes
☒	☐	Estimates of effect sizes (e.g. Cohen's *d*, Pearson's *r*), indicating how they were calculated

Our web collection on statistics for biologists contains articles on many of the points above.

Software and code

Policy information about availability of computer code

Data collection	Cryo-EM data were collected with EPU 3.
Data analysis	CryoEM data were analysed with WARP 1, RELION 3 and 4, cryoSPARC 4, Coot 0.9, Phenix 1.2, ISOLDE 1.6 and ChimeraX 1.7-1.10. All other data were analysed using FIJI with ImageJ 2.9, GraphPad Prism8 and R 4.0.<br>HT-Colabfold is free open-source software (MIT) and available at https://gitlab.com/BrenneckeLab/ht-colabfold.

For manuscripts utilizing custom algorithms or software that are central to the research but not yet described in published literature, software must be made available to editors and reviewers. We strongly encourage code deposition in a community repository (e.g. GitHub). See the Nature Research guidelines for submitting code & software for further information.

Data

Policy information about availability of data

All manuscripts must include a data availability statement. This statement should provide the following information, where applicable:

- Accession codes, unique identifiers, or web links for publicly available datasets
- A list of figures that have associated raw data
- A description of any restrictions on data availability

Three-dimensional cryo-EM density maps of the TREX–EJC–ALYREF complex, TREX-2M and UAP56–TREX-2M have been deposited into the Electron Microscopy Data Bank under the accession numbers EMD-18980 (Map-A) and EMD-18979 (Map-B), EMD-18977 (Map-C), EMD-18978 (Map-D) and EMD-18981 (Map-E) respectively. The coordinate files of the TREX–EJC–ALYREF, TREX-2M and UAP56–TREX-2M have been deposited into the Protein Data Bank under the accession numbers 8R7L, 8R7J, and 8R7K. The coordinate file of the TREX–mRNA complex was updated in the Protein Data Bank under the accession number 7ZNK. Proteomics data have been deposited to the ProteomeXchange Consortium via the PRIDE101 partner repository under the accession number PXD069399.

Field-specific reporting

Please select the one below that is the best fit for your research. If you are not sure, read the appropriate sections before making your selection.

☒ Life sciences ☐ Behavioural & social sciences ☐ Ecological, evolutionary & environmental sciences

For a reference copy of the document with all sections, see nature.com/documents/nr-reporting-summary-flat.pdf

Life sciences study design

All studies must disclose on these points even when the disclosure is negative.

Sample size	CryoEM data set sizes were chosen to achieve the desired resolution. All other sample sizes were chosen based on pilot experiments to detect biologically meaningful differences with statistical robustness.
Data exclusions	No data were excluded.
Replication	All experiments, except cryoEM data set collection and processing, were performed at least three times independently with comparable outcome. Sample preparation and the analysis were highly reproducible.
Randomization	For 3D refinement the cryo-EM data were split randomly into two halves for gold-standard FSC determination. All other experiments were not randomized but do contain appropriate controls.
Blinding	For the export tethering and RNA FISH experiments sample identities were revealed only after the analysis finished. For all other experiments Blinding is impractical. However, to minimize potential bias samples are always analyzed with the same pipeline and/or parameters for all samples and controls.

Reporting for specific materials, systems and methods

We require information from authors about some types of materials, experimental systems and methods used in many studies. Here, indicate whether each material, system or method listed is relevant to your study. If you are not sure if a list item applies to your research, read the appropriate section before selecting a response.

Materials & experimental systems

n/a	Involved in the study	
☐	☒	Antibodies
☐	☒	Eukaryotic cell lines
☒	☐	Palaeontology and archaeology
☒	☐	Animals and other organisms
☒	☐	Human research participants
☒	☐	Clinical data
☒	☐	Dual use research of concern

Methods

n/a	Involved in the study	
☒	☐	ChIP-seq
☒	☐	Flow cytometry
☒	☐	MRI-based neuroimaging

Antibodies

Antibodies used	Primary: anti-V5 antibody (Thermo Scientific, 37-7500, 2 µg ml-1 for GCI), anti-THOC2 (ab129485; Abcam; 1:1000), anti-UAP56 (ab181059; Abcam; 1:1000), anti-histone H3 (ab1791; Abcam; 1:1000), anti-UAP56 antibody (Cell Signaling Technology #47258; 1:1000), anti-GANP (ab113295; Abcam; 1:1000), anti-SARNP (PA5-56585; Invitrogen; 1:1000), anti-ALYREF (ab202894; Abcam; 1:1000), anti-V5 (2F11F7; Invitrogen; 1:1000), anti-Histone H3 (17168-1-AP; Proteintech; 1:1000), anti-GFP (CAS A11122, ThermoFisher, 1:1000), anti-EIF4A3 (AB180519, Abcam, 1:1000), anti-Histone H3-HRP (5192S, Cell Signalling Technologies; 1:1000) . Secondary: goat-anti-rabbit antibody coupled to HRP (Thermo Scientific 31466, diluted 1:5000), goat-anti-mouse antibody coupled to HRP (Thermo Scientific G-21040, diluted 1:5000)
Validation	All antibodies are validated by the manufacturer. In addition, anti-THOC, anti-SARNP, anti-UAP56, anti-GANP, anti-GFP and anti-V5 are validated assessing the detection of tagged proetins and/or the depletion of the respective protein in K562 cell lysate.

Eukaryotic cell lines

Policy information about cell lines

Cell line source(s)	Leukemia cell line K562 (source: ATCC)
Authentication	The cell line was authenticated by short tandem repeat analysis (see Muhar et al. 2018, Science, DOI: 10.1126/science.aao2793)

Mycoplasma contamination	The cell line was confirmed to be negative for mycoplasma.
Commonly misidentified lines (See ICLAC register)	No commonly misidentified cell line was used in this study.
